# Mental Health Challenges in Cancer Patients: A Cross-Sectional Analysis of Depression and Anxiety

**DOI:** 10.3390/cancers16162827

**Published:** 2024-08-12

**Authors:** Walid Shalata, Itamar Gothelf, Tomer Bernstine, Regina Michlin, Lena Tourkey, Sondos Shalata, Alexander Yakobson

**Affiliations:** 1 The Legacy Heritage Oncology Center & Dr. Larry Norton Institute, Soroka Medical Center, Ben-Gurion University, Beer-Sheva 84105, Israel; 2 Goldman Medical School, Faculty of Health Sciences, Ben-Gurion University of the Negev, Beer-Sheva 84105, Israel; 3 The Azrieli Faculty of Medicine, Bar-Ilan University, Safed 13115, Israel; 4 Nutrition Unit, Galilee Medical Center, Nahariya 22000, Israel

**Keywords:** cancer, depression, anxiety, mental health, patient-reported outcomes, psycho-oncology, metastasis

## Abstract

**Simple Summary:**

This research is suggested to address the significant psychological distress that cancer patients often experience during diagnosis and treatment, which can adversely affect their outcomes and care. Despite advancements in cancer treatments extending survival rates, the emergence of depression and anxiety as common comorbidities underscores the need for targeted interventions to improve patient well-being. The study aims to investigate the prevalence of depression and anxiety among cancer patients and identify some of the associated risk factors.

**Abstract:**

Advancements in cancer treatment and early detection have extended survival rates, transforming many cancers into chronic conditions. However, cancer diagnosis and treatment can trigger significant psychological distress, including depression and anxiety, impacting patient outcomes and care. This study aimed to examine the prevalence of and identify the risk factors for depression and anxiety among cancer patients. A cross-sectional study was conducted, including patients under the care of the oncology department at a tertiary medical center between June 2021 and October 2023. Depression and anxiety were assessed using the Patient-Reported Outcomes Measurement Information System (PROMIS) short forms. Logistic regression analysis identified risk factors for depression and anxiety. The study population included 159 patients, with 40.3% reporting worsening mental health, but only about half of them received therapy. Among the study participants, 22.6% experienced symptoms of depression and 30.2% experienced symptoms of anxiety. Single-cancer patients and those with metastases were at increased risk for depression, while those with a disease duration of more than a year and patients with female-specific cancer were more likely to experience anxiety. Given the high prevalence of mental health deterioration in cancer patients, closer monitoring and validated assessment tools are essential to improve depression and anxiety diagnosis and facilitate early interventions.

## 1. Introduction

Cancer diagnosis and treatment are life-changing experiences that demoralize cancer patients [1]. Recent advancements in cancer treatment represent the forefront of biomedical therapy, encompassing modalities such as chemotherapy, immunotherapy, targeted therapy, and radiotherapy. These approaches signify significant progress in improving patients’ quality of life and extending their survival rates [2,3]. Furthermore, enhancements in early cancer detection have contributed to an extended lifespan for individuals living with cancer, presenting a considerable global healthcare challenge. These advancements in the cancer field have resulted in a noteworthy outcome, wherein approximately fifty percent of individuals newly diagnosed with cancer can anticipate a minimum 10-year survival period [4]. This shift characterizes numerous cancer types as chronic conditions, thereby emphasizing the need for comprehensive, long-term management strategies.

In addition to the physical challenges posed by cancer and its treatments, receiving a cancer diagnosis is a profoundly stressful event and can be life-changing for the patient. The psychological response to this diagnosis can potentially trigger a range of serious health consequences, including the development of psychiatric disorders [5]. Among the most prevalent comorbid mental disorders are adjustment disorders (13%), followed by depression (11%) and anxiety disorders (10%) [6]. However, the relationship between mental health status and cancer-related complications is bidirectional. Over the past decade, there has been a rapid accumulation of evidence regarding the influence of mental disorders on tumor progression and cancer-related mortality [7]. Studies have found that psychiatric disorders adversely impact patient management and control, quality of life, duration of hospitalization, treatment compliance, and associated treatment expenses [8,9].

Prior research assessing psychological distress in cancer patients has revealed diverse prevalence rates, which varied between 5.0% and 49.0% depending on the disease stage, clinical setting, and treatment phase [10]. Another study found that approximately half of cancer patients report experiencing clinical levels of distress [11]. Variations across medical centers regarding the diagnosis and management of psychological distress within cancer care settings indicate a potential gap in awareness or recognition of these mental health issues [12]. Progress has been made in addressing the psychological needs of cancer patients. Oncologists are now receiving enhanced communication skills training to discuss psychological concerns with patients more effectively [13,14]. Additionally, screening systems have been developed to identify individuals experiencing depression and anxiety, taking into account various social factors [15,16]. However, to effectively implement these advances, oncology teams need to gain more exposure to these skills and screening systems and better understand the prevalence of depression and anxiety among their patients.

Evaluating depression and anxiety among cancer patients is vital to identifying those requiring support, additional evaluation, and follow-up interventions. One method of identifying such patients is through the administration of validated screening tests, as recommended by the National Comprehensive Cancer Network (NCCN) [17,18]. These screening tests use various questionnaires to detect patients with elevated levels of depression and anxiety symptoms, thereby indicating the potential need for additional psychological or psycho-oncological assistance and interventions [19]. Among these, the Patient-Reported Outcomes Measurement Information System (PROMIS) depression and anxiety short forms have gained increasing importance. These tools were designed to assess the severity of depression and anxiety symptoms through the patient’s self-reported experiences, aligning with psychological diagnostic methods rather than biomedical diagnostic criteria outlined in the DSM-5 [20].

Despite numerous research publications, the prevalence of depression and anxiety among clinically relevant cancer subgroups remains ambiguous, with varying estimates that are difficult to apply in clinical practice. Additionally, the association between cancer patients’ sociodemographic, oncologic, and psychiatric characteristics and their risk of developing depression and anxiety has not been adequately studied and remains unclear. We hypothesize that specific characteristics within these categories (e.g., marital status and gender) are associated with an increased risk of depression and anxiety among cancer patients. Therefore, the objectives of this study are: (1) to evaluate the prevalence of depression and anxiety among cancer patients in Israel; and (2) to explore the relationships between various sociodemographic, oncologic, and psychiatric characteristics and the risk of depression and anxiety. Addressing this knowledge gap is essential for raising awareness of these mental health issues and facilitating early identification and personalized interventions. This approach can promote holistic disease management, improve the overall quality of life, and reduce mortality for cancer patients.

## 2. Materials and Methods

### 2.1. Study Design

This is a cross-sectional study conducted at the Soroka University Medical Center (SUMC) in Beer-Sheva, Israel, between June 2021 and October 2023. SUMC serves as the principal hospital and the sole specialized tertiary care facility in Israel’s southern region, an area that encompasses approximately 60% of the nation’s territory [21]. It delivers a full spectrum of clinical management and oncological services, addressing the needs of both adult and pediatric patients within Beer-Sheva and its adjacent areas.

### 2.2. Sample Characteristics

From June 2021 through October 2023, all cancer patients under the care of the oncology department at SUMC were invited by their physicians to participate in a cross-sectional study. Patients who consented to participate in the study were provided with a comprehensive explanation of its content and purposes. Participants were notified that their assent to engage in the study would be acknowledged with their written consent. Subsequently, they were requested to complete a detailed questionnaire as part of the study protocol.

The inclusion criteria for the study were: (a) cancer patients under the care of the oncology department at SUMC undergoing treatment modalities (immunotherapy, chemotherapy, chemo-immunotherapy, or radiotherapy); (b) patients capable of reading in Hebrew, Arabic, English, and Russian and willing to provide informed consent; (c) patients aged 18 years or older.

### 2.3. Assessment

Patients completed self-administered, anonymous written questionnaires. The responses were securely stored in a locked facility at SUMC. Subsequently, a separate research member processed the questionnaire data using a computerized system. The questionnaires were composed of five modules: (a) Sociodemographic characteristics included age, gender, ethnicity, marital status. (b) Oncologic characteristics included cancer metastasis (yes/no), disease duration, cancer therapy and family history of malignancy (yes/no). (c) Psychiatric characteristics included past mental health disorder, past anti-depressant treatment (yes/no), type of treatment, family history of diagnosed mental health disorder (yes/no), is the patient feeling mental health worsening (yes/no), currently using pharmacology therapy (yes/no), and current psychiatric/psychologic support (yes/no). (d) The evaluation of anxiety was performed using the seven-question version of the PROMIS Emotional Distress–Anxiety short form, translated into Hebrew, Arabic, English, and Russian. This validated assessment tool is a widely recognized self-administered instrument formulated by the National Institutes of Health for quantifying anxiety symptoms [22,23]. Each question was rated from 1, indicating ‘never’, to 5, signifying ‘always’ [24,25]. (e) Depression was assessed using the eight-question version of the PROMIS Emotional Distress–Depression short form, a version specifically designed to screen for depression. This form was also translated into Hebrew, Arabic, English, and Russian. Similarly, each question rated on a scale from 1 (‘never’) to 5 (‘always’), following a consistent rating pattern [26].

### 2.4. Methods

The PROMIS depression form included 8 items, each scored from 1 to 5. The scores were summed to obtain a raw score ranging from 8 to 40, which was then converted into a T-score ranging from 37.1 to 81.1. Similarly, the PROMIS anxiety form included 7 items, each scored from 1 to 5, resulting in a raw score ranging from 7 to 35 [27]. This raw score was converted into a T-score ranging from 36.3 to 82.7. For both forms, a T-score of less than 55 indicated no to slight depression or anxiety, 55–59.9 indicated mild depression or anxiety, 60–69.9 indicated moderate depression or anxiety, and 70 or above indicated severe depression or anxiety.

### 2.5. Statistical Analysis

Descriptive statistics were employed to summarize the patients’ sociodemographic, oncologic, and psychiatric characteristics. Continuous data were presented as mean ± standard deviation (SD), while categorical variables were reported as frequencies (percentages). Logistic regression was utilized to evaluate the primary hypotheses that certain sociodemographic, oncologic, and psychiatric factors are associated with the presence of depression and anxiety. The variables included in the regression model were selected based on their established relevance to depression and anxiety as documented in the existing literature. This method estimated odds ratios (ORs) and 95% confidence intervals (CIs) for these associations. Logistic regression was preferred for its ability to handle binary dependent variables and adjust for multiple confounders, providing a distinct advantage when addressing categorical outcomes. The cut-off points for depression and anxiety were determined according to DSM-level 2 criteria, as detailed in the Section 2.4. For both depression and anxiety, a T-score of 55 and above was used as the threshold, reflecting the standard criterion commonly used to indicate clinically significant symptoms. Statistical analysis was performed using SPSS software (version 29). A two-sided *p*-value of less than 0.05 was considered statistically significant.

## 3. Results

During the recruitment period from June 2021 and October 2023, 159 patients provided their consent to participate in the study. Table 1 presents the background characteristics of the study participants, whose median age was 67.0 with an interquartile range (IQR) of 11. The majority of the participants were males (62.3%, with a median age of 69 and an IQR of 8), whereas the female participants had a mean age of 64.65 ± 10.57 years. Table 2 provides a detailed analysis of these descriptive statistics. In total, 57.2% identified as Jewish, 19.5% as Arab, and 23.3% as of European descent, with 66% of the participants being married. Among the participants, 69.8% (*n* = 111) reported having metastatic cancer, with most treated either with chemotherapy alone (*n* = 69, 43.4%) or a combination of immunotherapy and chemotherapy (*n* = 47, 29.6%). Approximately half of the patients (*n* = 81, 50.9%) had a disease duration of more than one year, and 45.3% (*n* = 72) reported a family history of malignancy.

Among the 159 patients, 10.1% (*n* = 16) reported having received past mental health treatment, primarily medications (*n* = 10, 6.3%). Additionally, 3.8% (*n* = 6) mentioned a family history of diagnosed mental health disorders. Notably, 40.3% (*n* = 64) of all participants reported a subjective deterioration of their mental status, but only 6.3% (*n* = 10) were currently undergoing medical therapy, whereas 18.9% (*n* = 30) were receiving psychiatric or psychological support.

Table 3 below provides a breakdown of the prevalence of anxiety and depressive symptoms among patients, categorized by severity. Anxiety symptoms, as assessed using the PROMIS Emotional Distress–Anxiety short form, were identified in 30.2% (*n* = 48) of the patients. Among these, 15.1% (*n* = 24) reported mild anxiety, 13.2% (*n* = 21) reported moderate anxiety, and 1.9% (*n* = 3) reported severe anxiety. By comparison, depressive symptoms, evaluated using the PROMIS Emotional Distress–Depression short form, were less prevalent, affecting 22.6% (*n* = 36) of the patients. Within this group, 13.2% (*n* = 21) experienced mild depression, 7.5% (*n* = 12) experienced moderate depression, and 1.9% (*n* = 3) experienced severe depression. Table 4 provides further descriptive statistics related to the PROMIS form.

The prevalences of depression and anxiety, stratified by the type and severity of cancer, are presented in Table 5. Lung cancer was the most prevalent type of cancer among the patients, accounting for 23.3% (*n* = 37) of cases. Within this subgroup, 27% (*n* = 10) of patients experienced anxiety symptoms, while 21.6% (*n* = 8) exhibited symptoms of depression. Similarly, colorectal cancer was diagnosed in 17.0% (*n* = 27) of the participants, with 18.5% (*n* = 5) of these patients describing anxiety and 29.6% (*n* = 8) reporting depression. Furthermore, skin and breast cancers constituted 15.7% (*n* = 25) and 13.2% (*n* = 21) of the cases, respectively. Notably, 38.9% (*n* = 8) of breast cancer patients and 28% (*n* = 7) of skin cancer patients reported anxiety symptoms.

The multivariable logistic regression analysis, as detailed in Table 6, examined various characteristics that could impact the diagnosis of anxiety and depression based on the PROMIS questionnaires. The findings indicate that patients who were single (OR = 10.89, *p* = 0.002, 95% CI 2.44–48.64), those with metastatic disease (OR = 3.66, *p* = 0.033, 95% CI 1.11–12.01), and those with past mental treatment history (OR = 4.30, *p* = 0.033, 95% CI 1.12–16.48) faced a significantly increased risk of reporting depression. Additionally, the multivariable logistic regression analysis for anxiety revealed that patients with a disease duration of more than one year had a significantly increased risk of reporting anxiety symptoms (OR = 2.65, *p* = 0.022, 95% CI 1.15–6.08), while patients with female-specific types of cancer (OR = 7.78, *p* = 0.045, 95% CI 1.05–57.96) were at a significantly heightened risk for reporting anxiety symptoms and marginally significantly more at risk for reporting symptoms of depression (OR = 8.53, *p* = 0.071, 95% CI 0.83–87.41). Supplementary S1 illustrates depression/anxiety among cancer patients across various demographic and clinical characteristics.

## 4. Discussion

This study aimed to investigate the prevalence of mental health conditions, specifically depression and anxiety, across clinically relevant cancer subgroups and to identify the risk factors associated with these conditions. An assessment of depression and anxiety symptoms was conducted using the PROMIS self-report questionnaires. Patients scoring above the threshold (T-score of 55 or higher) were considered within the clinical range for symptoms. Clinical symptoms of depression and anxiety stem from a variety of factors, including medical, psychological, and cultural. The study was composed of several key sections: (a) characterization of the patient population; (b) the prevalence and severity of depression and anxiety symptoms, stratified by multiple cancer types; and (c) examination of various risk factors contributing to the development of depression and anxiety in cancer patients.

In this study, 22.6% of cancer patients experienced depression symptoms, while 30.2% experienced anxiety. A comparable study conducted in Alabama, USA, utilizing the PROMIS depression form reported a depression prevalence of 25.9% [28]. Another study conducted in California, USA, reported markedly higher rates than those commonly observed in the literature [29], with 47% of cancer patients experiencing symptoms of depression and 45% experiencing symptoms of anxiety [30]. It is important to note that these studies evaluated the prevalence of depression and anxiety using the PROMIS questionnaire among specific cancer subtypes, while PROMIS data regarding a wider range of cancer subtypes remain limited. Our study examined individuals at risk for mental health issues and assessed whether they received appropriate treatment. Among the 40.3% of cancer patients who reported a deterioration in their mental status, only 6.3% were currently taking anti-depressant medications. The alarmingly low rates of patients receiving anti-depressant medications highlight a significant and concerning issue, suggesting that cancer patients may not be receiving the necessary therapy, which could adversely impact their quality of life. This finding aligns with a US study that revealed that only 12% of those diagnosed with depression received antidepressant medications, and a mere 5% reached a mental health counsellor [31]. This discrepancy may stem from insufficient mental health evaluations. Following a cancer diagnosis, patients must receive multidisciplinary support, including mental health care, to monitor and address potential symptoms of anxiety and depression [32,33]. Another factor contributing to the under-diagnosis of depression and anxiety is that oncologists diagnose mild to moderate depressive and anxiety symptoms in only one-third of patients who exhibit these symptoms and tend to underestimate the severity of these symptoms compared to patients who are more severely affected [34]. It should also be considered that once medical therapy is initiated, close follow-up is essential to evaluate the effectiveness of the treatment, monitor patient compliance, and assess for potential side effects and drug interactions [35].

Our study found different risk factors for depression and anxiety. The results indicated that single patients were more prone to depression compared to their married counterparts. This finding is consistent with a study by Aizer et al. that reported similar outcomes [36]. Our results indicate that single individuals that were diagnosed with cancer exhibited significantly higher levels of depression and anxiety compared to those in other relationship statuses. We hypothesize that this may be due to increased concerns about rejection and difficulties in forming new relationships. Extended periods of singlehood can lead to heightened caution when interacting with potential partners, which may adversely affect mental health. Long-term singlehood can foster feelings of loneliness, isolation, and social exclusion, potentially contributing to depression and anxiety. Additionally, societal pressures and personal beliefs about relationships might diminish self-esteem or self-worth. Prolonged singlehood may also undermine confidence in dating, further exacerbating social anxiety. Thus, the increased susceptibility to depression among single individuals may reflect broader issues related to social support and societal structures especially after a diagnosis of cancer. Married individuals often benefit from emotional and practical support from their partners, which can buffer against the psychological stress of cancer diagnosis and treatment. In contrast, single patients may lack immediate access to such support, potentially leading to higher emotional burdens and mental health challenges. This aligns with the understanding that social support networks are critical to mental health outcomes. Additionally, single patients demonstrate poorer adherence to the various and demanding treatments compared to their married counterparts throughout the disease progression [37]. This discrepancy may arise from the absence of a supportive partner who can assist in managing the logistical and emotional aspects of treatment adherence. The relationship between therapy adherence and depression has been explored in various studies, indicating that a lack of support can lead to higher depression rates [38,39]. Therefore, oncologists and mental health providers should adopt a holistic approach to assessing cancer patients for depression and anxiety, considering both individual circumstances and broader social contexts, ultimately improving patients’ well-being and quality of life.

In addition to marital status, metastasis was also found to be associated with an increased risk of depression in our study. This association is supported by the literature and can be attributed to several factors [40,41]. Metastatic disease is often linked with a poorer prognosis and lower survival rates, which can lead to heightened feelings of hopelessness, despair, and depression. Moreover, metastatic cancer is associated with an increased symptom burden, chronic pain, and severe physical limitations, all of which are known risk factors for depression [42]. The aggressive treatments required for metastatic cancer usually result in severe side effects that contribute to both physical discomfort and emotional distress, further exacerbating the risk of depression in these patients. 

Furthermore, our data showed that patients with a history of antidepressant use are at an increased risk of depression following a cancer diagnosis. This finding is plausible, as these patients likely have a history of depression and a predisposition to depressive episodes. The emotional and physical burdens of a cancer diagnosis and treatment can disrupt previously stable remission of depressive symptoms, leading to psychological imbalance. This disruption can reactivate underlying depressive tendencies, exacerbating the patient’s mental health condition [43].

A disease duration of more than a year was linked in our study to an increased risk of anxiety, contrary to the findings of Krebber et al. [44]. This discrepancy may be due to the relatively high proportion of patients (96.2%) in our study who were still undergoing cancer therapy, with almost 70% receiving chemotherapy. Prolonged cancer therapy, particularly chemotherapy, can lead to long-term emotional exhaustion and overwhelm the patient’s coping mechanisms. Additionally, as cancer progresses, the physical symptoms and side effects of treatments can become more severe, resulting in increased physical pain and discomfort, further contributing to the heightened risk of anxiety [45].

Our study also found that the type of cancer impacts the development of anxiety. Specifically, we found that patients with female-specific cancers, predominantly breast cancer, had a higher probability of reporting anxiety symptoms compared to those with other types of cancer, and also a trend for higher probability of reporting symptoms of depression. Although some types of cancer may require more intensive treatments than breast cancer, this does not appear to be the predominant factor influencing anxiety levels. Factors such as coping strategies, social support, and social reintegration play crucial roles in the development of depression and anxiety [46]. A possible explanation for this finding may be that the emotional and psychological burden of a breast cancer diagnosis, along with concerns about body image, both contribute to heightened depression and anxiety levels in these patients [47].

Another explanation for this finding, supported by the existing literature [48,49], may be that women often employ emotion-focused coping strategies [50]. These strategies make them more acutely aware of their condition and personal distress. In contrast, men tend to adopt more restrained and self-contained attitudes, leading to fewer complaints and less emotional sharing. While the psychological challenges and coping mechanisms utilized by women with female-specific cancers are not yet fully understood and warrant further research, it is essential to evaluate sub-groups of patients, particularly women, for mental health conditions, specifically depression and anxiety. Following this evaluation, patients should receive appropriate psychological support and medical treatment.

In this study, we compared the prevalence of depression and anxiety among three ethnic groups: Jewish, Arabs, and European descent women. Interestingly, our findings did not reveal significant differences in the incidence of these mental health issues among the groups, suggesting that the overall burden of these mental health morbidities is similarly distributed despite their diverse backgrounds. In an 18-center meta-analysis in the USA, Hispanic cancer patients reported higher levels of depression compared to other ethnicities [51]. However, the prevalence of depression and anxiety among Jewish and Arab cancer patients has not yet been studied. The Arabs are a traditionally nomadic Muslim ethnic group characterized by a distinct cultural heritage and lifestyle [52]. Despite facing unique socio-economic challenges, such as limited access to healthcare and lower socio-economic status, the incidence of these mental health morbidities did not significantly differ from other groups. These findings highlight the need for further study of these factors and a deeper examination of the varied characteristics among different ethnicities.

Indeed, we did not find significant gender differences in our study in the rates of anxiety and depression symptoms. A recent meta-analysis found that, overall, female cancer patients reported significantly higher levels of depression compared to their male counterparts, while males reported significantly greater levels of anxiety than females [53]. Yet, the findings were inconsistent among studies. Thus, further research is needed in order to conclude whether there are indeed gender differences in the rates of depression and anxiety in cancer patients.

To our knowledge, this is the first study to utilize the PROMIS anxiety and depression questionnaires, well-established and validated assessment tools, to evaluate the prevalence and severity of anxiety and depression across various types of cancer in a diverse ethnic population. Additionally, this study aimed to identify risk factors associated with anxiety and depression in a varied cancer patient population, without restrictions on age, ethnicity, gender, family status, type of treatment, or type of cancer. 

However, this study has several limitations. First and foremost, the relatively small sample size of our study may limit the generalizability of our findings, potentially limiting our ability to adequately represent the broader population of cancer patients. Specifically, the heterogeneity of the study population and the limited numbers within certain cancer type subgroups hinder our ability to detect significant associations and draw robust conclusions. Yet, to our knowledge, this is the first study from Israel reporting on the rates of depression and anxiety in adults with various types of cancers. Additionally, due to the cross-sectional design, it is not possible to establish causality between the different variables; it can only demonstrate associations between them. Furthermore, the study lacks measures of anxiety and depression at different time points, which could help to evaluate the mental health status throughout disease progression. Such a follow-up could also aid in detecting patients who are not receiving proper therapy or any therapy at all. Moreover, anxiety and depression were assessed using the PROMIS questionnaire, which may not provide a comprehensive assessment of the patient’s full mental status, thus limiting our ability to determine a patient-tailored intervention based on the study outcomes. In addition, the questionnaire used in this study did not account for the number of divorces within the marital status category of participants, nor did it inquire about the specific generation of family history of malignancy, thereby limiting our ability to ascertain the extent of familial malignancy history. Finally, self-filled questionnaires can be a limitation due to potential biases such as self-reporting bias and recall bias, which can affect the accuracy of the data. Future studies should aim to include larger sample sizes and incorporate continuous follow-up with multiple evaluation points.

## 5. Conclusions

This study highlights the high prevalence of mental health deterioration among cancer patients and identifies key risk factors for depression and anxiety. The findings suggest that closer monitoring of mental health and the use of validated assessment tools in cancer patients can lead to better diagnosis and, as a result, timely and effective interventions. It is important for clinicians to be aware of these outcomes and provide suitable psycho-oncologic support for their patients. Future research should focus on large-scale longitudinal studies to better understand the course of mental health symptoms in cancer patients and to identify the most critical periods for intervention. Additionally, there is a need to explore the impact of tailored mental health interventions and support systems on improving patient outcomes. Physicians should consider routinely using short self-report mental health questionnaires, such as the PROMIS, for screening anxiety and depression symptoms in patients with cancer. By prioritizing mental health, clinicians can significantly enhance the overall well-being and quality of life of their patients.

## Figures and Tables

**Table 1:** The baseline characteristics of the study population (*n* = 159).

Characteristics		Median (Interquartile Range)
Age (years)		67.0 (11)
Gender		
	Male Female	69.0 (8) Mean ± SD (range) 64.65 ± 10.57 (44–87)
		Frequencies (percentage)
	Female	60 (37.7)
	Male	99 (62.3)
Ethnicity		
	Jewish	91 (57.2)
	Arab	31 (19.5)
	European descent	37 (23.3)
Marital status		
	Married	105 (66.0)
	Divorced	25 (15.7)
	Widowed	14 (8.8)
	Single	11 (6.9)
	In relationship	4 (2.5)
Cancer metastasis		
	Yes	111 (69.8)
	No	48 (30.2)
Disease duration		
	<1 month	3 (1.9)
	1–3 month	19 (11.9)
	3–6 month	29 (18.2)
	6–9 month	18 (11.3)
	9–12 month	9 (5.7)
	>1 year	81 (50.9)
Cancer therapy		
	Immunotherapy	38 (23.9)
	Chemotherapy	69 (43.4)
	Combination of immunotherapy and chemotherapy	47 (29.6)
	Radiotherapy	5 (3.1)
Family history of malignancy		
	Yes	72 (45.3)
	No	87 (54.7)
Past mental health disorder		
	Yes	7 (4.4)
	No	152 (95.6)
Past antidepressant treatment		
	Yes	16 (10.1)
	No	143 (89.9)
Type of antidepressant treatment		
	Psychotherapy	2 (1.3)
	Medications	10 (6.3)
	Combination	4 (2.5)
Family history of diagnosed mental health disorder		
	Yes	6 (3.8)
	No	153 (96.2)
Are you feeling mental health worsening?		
	Yes	64 (40.3)
	No	95 (59.7)
Currently using pharmacology therapy		
	Yes	10 (6.3)
	No	149 (93.7)
Current psychiatric/psychological support		
	Yes	30 (18.9)
	No	129 (81.1)

**Table 2:** Descriptive statistics of age by gender.

	*N*	Mean	Median	Standard Deviation	Variance	Skewness Coefficient
Age (Total)	159	65.75	67	10.99	120.72	−1.180
Female	60	64.65	64.0	10.57	111.62	0.210
Male	99	66.41	69.0	11.24	126.25	−1.918

**Table 3:** Prevalence of depression and anxiety among the patients, stratified by severity (*n* = 159).

Severity of Depression	Prevalence (Percentage)
Normal	123 (77.4)
Mild depression	21 (13.2)
Moderate depression	12 (7.5)
Severe depression	3 (1.9)
**Severity of anxiety**	**Prevalence (percentage)**
Normal	111 (69.8)
Mild anxiety	24 (15.1)
Moderate anxiety	21 (13.2)
Severe anxiety	3 (1.9)

**Table 4:** Descriptive statistics of PROMIS T-score.

PROMIS T-Score	*N*	Mean	Median	Standard Deviation	Variance	Skewness Coefficient
Depression	159	46.56	46.20	9.16	83.90	0.61
Anxiety	159	48.64	48.40	9.99	99.88	0.26

**Table 5:** Prevalence of depression and anxiety stratified by type of cancer and severity.

	Liver Cancer	Renal Cancer	Pancreas Cancer	Brain Cancer	Skin Cancer	Sarcoma Cancer	Prostate Cancer	Neck Cancer	Colorectal Cancer	Stomach Cancer	Uterine Cancer	Lung Cancer	Breast Cancer	Ovarian Cancer	Bladder Cancer
**Depression severity**															
Normal	5 (100.0)	3 (60.0)	3 (42.9)	0	22 (88.0)	3 (50.0)	6 (85.7)	0	22 (81.5)	7 (87.5)	0	29 (78.4)	16 (76.2)	5 (100.0)	2 (100.0)
Mild depression	0	1 (20.0)	2 (28.6)	1 (100.0)	1 (4.0)	1 (16.7)	1 (14.3)	1 (50.0)	3 (11.1)	0	0	7 (18.9)	3 (14.3)	0	0
Moderate depression	0	1 (20.0)	2 (28.6)	0	2 (8.0)	1 (16.7)	0	1 (50.0)	2 (7.4)	0	1 (100.0)	1 (2.7)	1 (4.8)	0	0
Severe depression	0	0	0	0	0	1 (16.7)	0	0	0	1 (12.5)	0	0	1 (4.8)	0	0
**Total**	5 (3.1)	5 (3.1)	7 (4.4)	1 (0.6)	25 (15.7)	6 (3.8)	7 (4.4)	2 (1.3)	27 (17.0)	8 (5.0)	1 (0.6)	37 (23.3)	21 (13.2)	5 (3.1)	2 (1.3)
**Anxiety severity**															
Normal	5 (100.0)	4 (80.0)	4 (57.1)	0	18 (72.0)	3 (50.0)	6 (85.7)	0	19 (70.4)	6 (75.0)	0	27 (73.0)	13 (61.9)	4 (80.0)	2 (100.0)
Mild anxiety	0	0	1 (14.3)	1 (100.0)	3 (12.0)	1 (16.7)	1 (14.3)	2 (100.0)	4 (14.8)	0	0	7 (18.9)	3 (14.3)	1 (20.0)	0
Moderate anxiety	0	1 (20.0)	2 (28.6)	0	3 (12.0)	1 (16.7)	0	0	4 (14.8)	2 (25.0)	1 (100.0)	3 (8.1)	4 (19.0)	0	0
Severe anxiety	0	0	0	0	1 (4.0)	1 (16.7)	0	0	0	0	0	0	1 (4.8)	0	0
**Total**	5 (3.1)	5 (3.1)	7 (4.4)	1 (0.6)	25 (15.7)	6 (3.8)	7 (4.4)	2 (1.3)	27 (17.0)	8 (5.0)	1 (0.6)	37 (23.3)	21 (13.2)	5 (3.1)	2 (1.3)

**Table 6:** Multivariable logistic regression analysis of sociodemographic, oncologic, and psychiatric characteristics and their associations with the risk of developing depression and anxiety.

Variable	Odds Ratio for Depression; CI 95%	*p*-Value for Depression	Odds ratio for Anxiety; CI 95%, *p*-Value	*p*-Value for Anxiety
Gender				
Female (reference)	1		1	
Male	1.56; 0.48–5.05	0.462	2.55; 0.86–7.59	0.093
Marital status				
Married (reference)	1		1	
Divorced	1.28; 0.39–4.20	0.688	1.97; 0.71–5.48	0.195
Widowed	0.88; 0.16–4.94	0.881	0.25; 0.03–2.23	0.214
Single	10.89; 2.44–48.64	0.002	2.89; 0.71–11.71	0.137
In relationship	0;	0.999	1.51; 0.13–17.37	0.740
Duration of disease				
<1 year (reference)	1		1	
>1 year	1.41; 0.56–3.54	0.464	2.65; 1.15–6.08	0.022
Metastasis				
No (reference)	1		1	
Yes	3.66; 1.11–12.01	0.033	1.17; 0.49–2.79	0.722
Cancer therapy				
Immunotherapy (reference)	1		1	
Chemotherapy	1.27; 0.31–5.26	0.742	0.46; 0.12–1.67	0.236
Combination of immunotherapy and chemotherapy	0.54; 0.11–2.51	0.428	0.26; 0.06–1.03	0.056
Radiotherapy	2.80; 0.26–29.75	0.394	1.06; 0.11–9.91	0.962
Ethnicity				
Jewish (reference)	1		1	
Arab	1.30; 0.44–3.85	0.634	1.75; 0.63–4.88	0.284
European descent	0.88; 0.27–2.87	0.834	1.12; 0.42–2.97	0.819
Age				
Less than 65 (reference)	1		1	
65 years and above	0.75; 0.30–1.90	0.549	0.47; 0.20–1.07	0.072
Type of cancer				
Skin (reference)	1		1	
Colon	3.10; 0.38–25.69	0.294	3.21; 0.57–17.78	0.182
Lung	5.0; 0.72–34.92	0.104	2.71; 0.59–12.52	0.202
Female-specific (breast, ovarian, uterine)	8.53; 0.83–87.41	0.071	7.78; 1.05–57.96	0.045
Other	6.40; 0.94–43.72	0.058	2.69; 0.56–12.88	0.217
Past mental treatment				
No (reference)	1		1	
Yes	4.30; 1.12–16.48	0.033	2.10; 0.61–7.25	0.239

## Data Availability

The data either reside within the article itself or can be obtained from the authors upon making a reasonable request.

## Supplementary Materials

The following supporting information can be downloaded at: https://www.mdpi.com/article/10.3390/cancers16162827/s1, Supplementary S1: Depression and Anxiety Among Cancer Patients by Various Demographic and Clinical Factors.
