# Supplementary material for: Mental Health Challenges in Cancer Patients: A Cross-Sectional Analysis of Depression and Anxiety
Source: Cancers (Basel). 2024 Aug 12;16(16):2827. doi: 10.3390/cancers16162827 (PMC11352929; doi:10.3390/cancers16162827)
Supplement: Supplementary file 1 [file cancers-16-02827-s001.zip › cancers-3157849-supplementary.pdf]

Supplementary S1: Depression and Anxiety Among Cancer Patients by Various Demographic and Clinical Factors

Depression

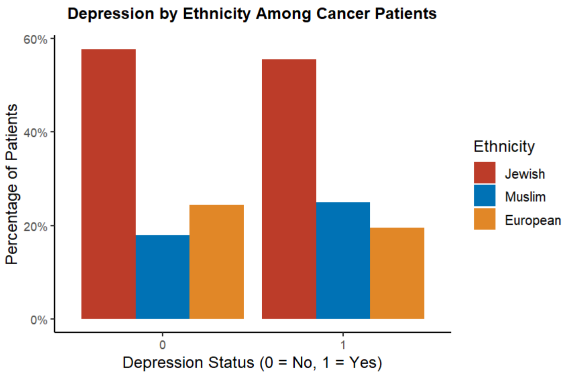

(I)

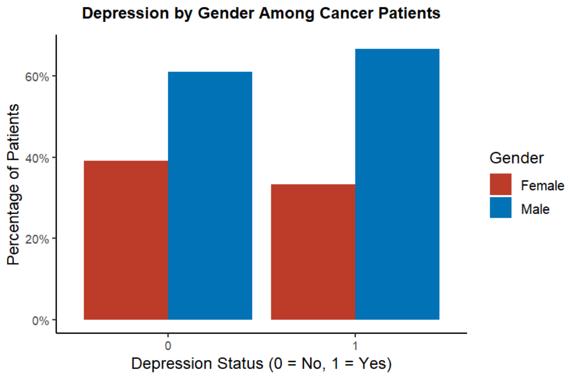

(II)

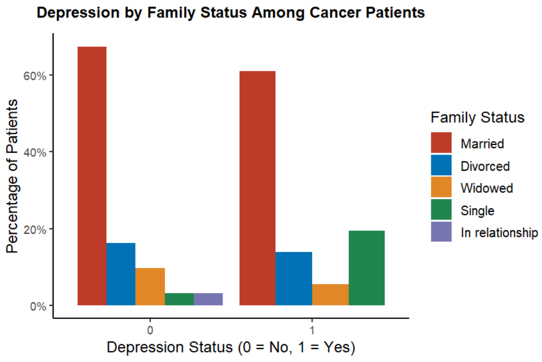

(III)

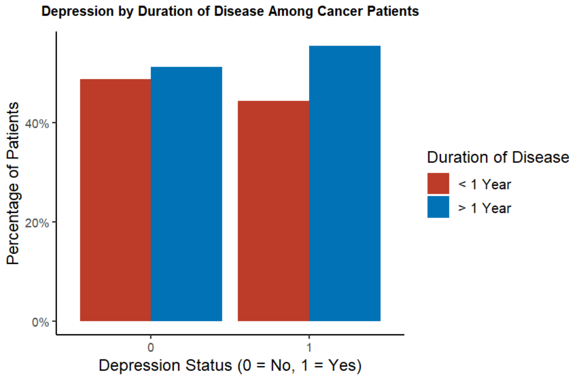

(IV)

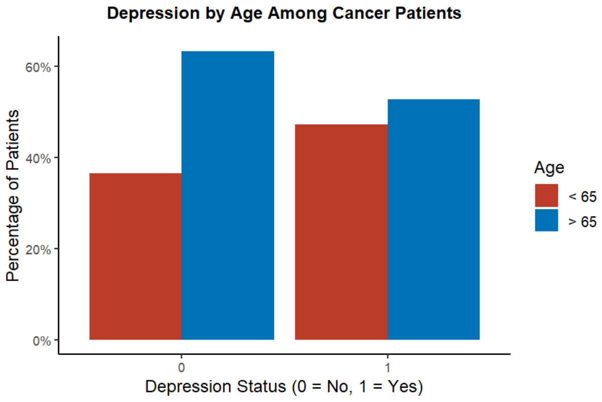

(V)

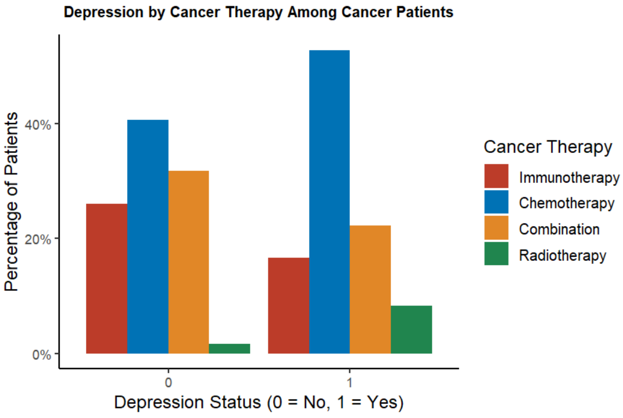

(VI)

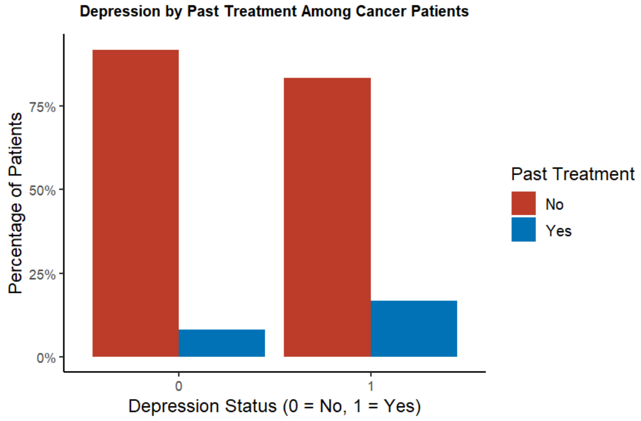

(VII)

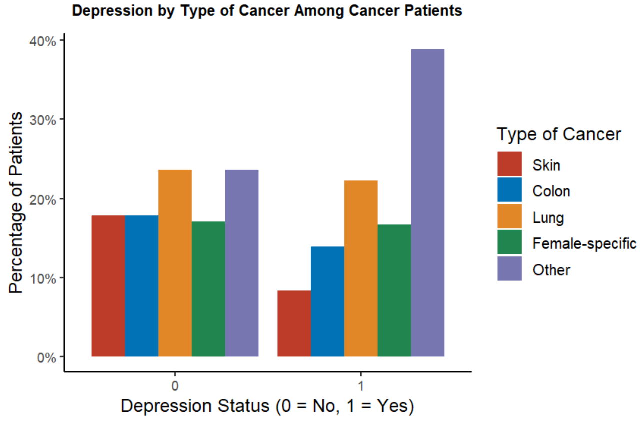

(VIII)

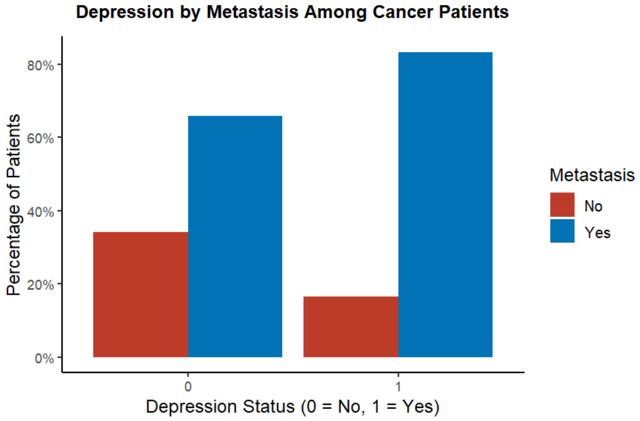

(IX)

$$\log\left(\frac{P(Y=1)}{P(Y=0)}\right) = -5.765 + 1.296 \times \text{metastasis} + 2.388 \times \text{single} + 1.458 \times \text{past mental treatment}$$

Anxiety

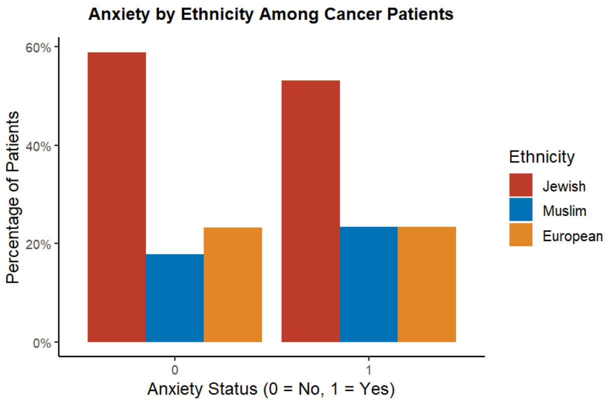

(I)

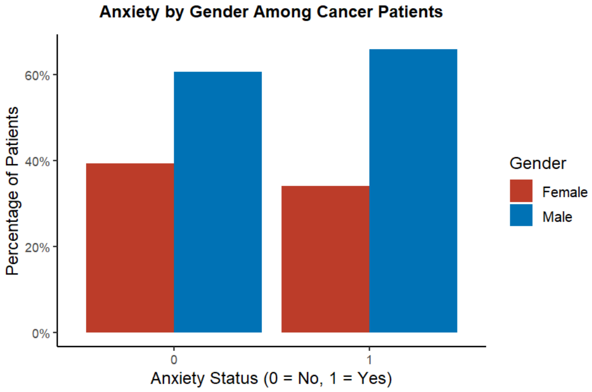

(II)

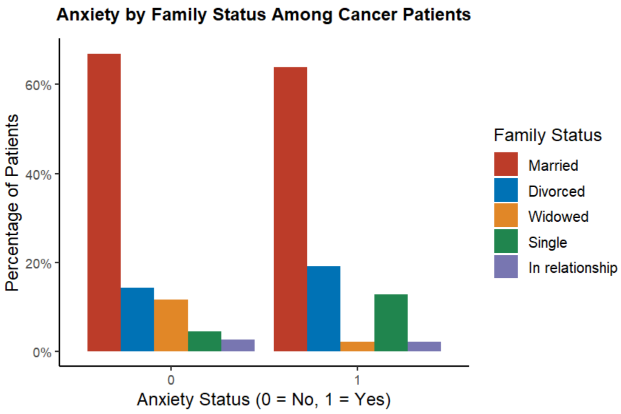

(III)

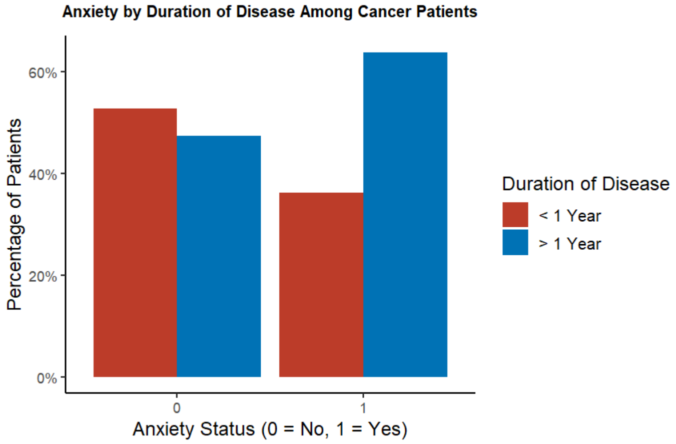

(IV)

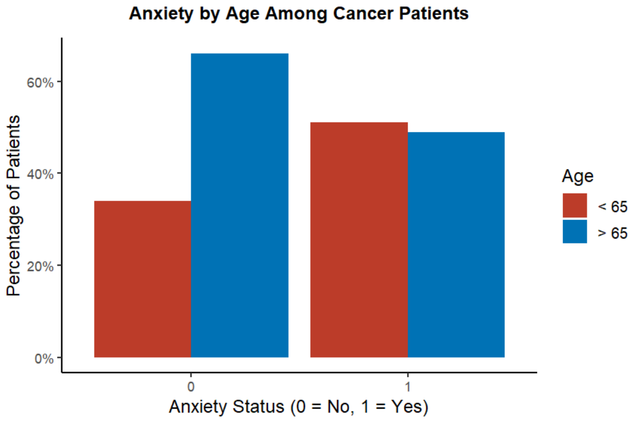

(V)

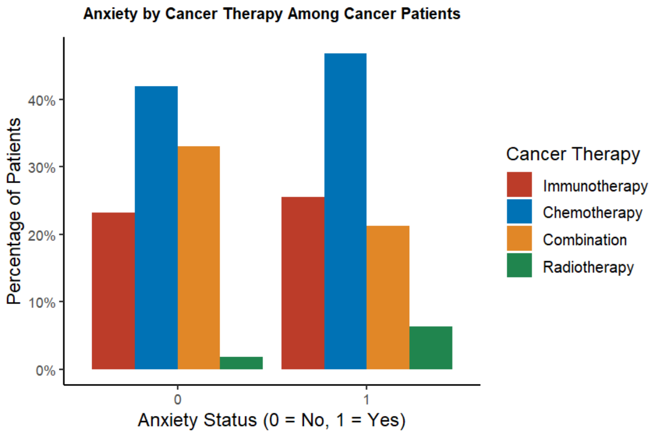

(VI)

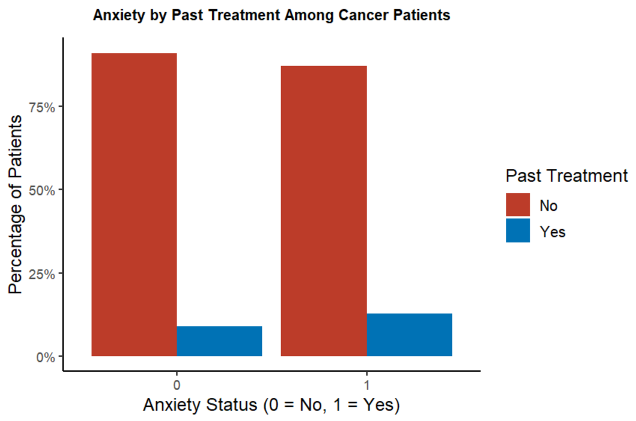

(VII)

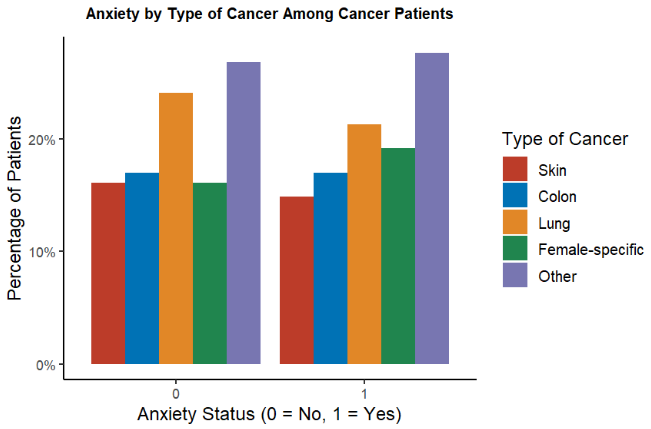

(VIII)

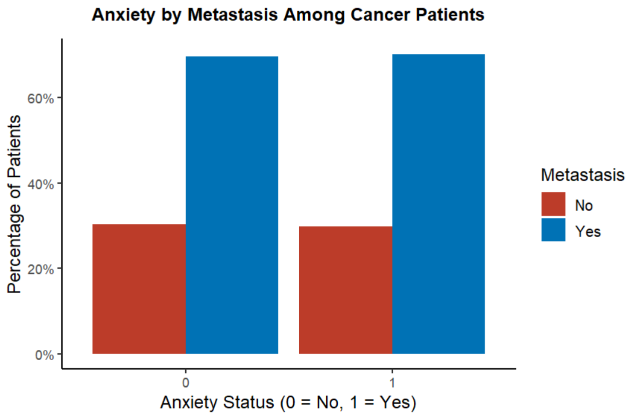

(IX)

$$\log\left(\frac{P(Y=1)}{P(Y=0)}\right) = -2.534 + 2.052 \times \text{female-specific type} + 0.974 \times \text{disease duration}\,(>1 \text{ year})$$
